# An epitope-optimized human H3N2 influenza vaccine induces broadly protective immunity in mice and ferrets

**DOI:** 10.1038/s41541-022-00492-y

**Published:** 2022-06-23

**Authors:** Brianna L. Bullard, Jennifer DeBeauchamp, Matthew J. Pekarek, Erika Petro-Turnquist, Peter Vogel, Richard J. Webby, Eric A. Weaver

**Affiliations:** 1 School of Biological Sciences, Nebraska Center for Virology, University of Nebraska, Lincoln, NE 68503 USA; 2 Department of Infectious Diseases, St. Jude Children’s Research Hospital, Memphis, TN 38105 USA; 3 Department of Pathology, St. Jude Children’s Research Hospital, Memphis, TN 38105 USA

**Keywords:** Vaccines, Infectious diseases

## Abstract

There is a crucial need for an improved H3N2 influenza virus vaccine due to low vaccine efficacy rates and increased morbidity and mortality associated with H3N2-dominated influenza seasons. Here, we utilize a computational design strategy to produce epitope-optimized, broadly cross-reactive H3 hemagglutinins in order to create a universal H3N2 influenza vaccine. The Epigraph immunogens are designed to maximize the viral population frequency of epitopes incorporated into the immunogen. We compared our Epigraph H3 vaccine to the traditional egg-based inactivated influenza vaccine from 2018–19, FluZone. Epigraph vaccination-induced stronger cross-reactive antibody responses than FluZone against 18 H3N2 viruses isolated from 1968 to 2019 in both mice and ferrets, with protective hemagglutination inhibition titers against 93–100% of the contemporary H3N2 strains compared to only 27% protection measured from FluZone. In addition, Epigraph vaccination-induced strong cross-reactive T-cell immunity which significantly contributes to protection against lethal influenza virus infection. Finally, Epigraph vaccination protected ferrets from influenza disease after challenge with two H3N2 viruses. The superior cross-reactive immunity induced by these Epigraph immunogens supports their development as a universal H3N2 influenza vaccine.

## Introduction

Annually, seasonal influenza epidemics infect up to one billion people globally and result in 3–5 million severe cases with as many as 650,000 deaths^1–3^. Symptoms typically include fever, headache, myalgia, and respiratory distress and last between 5 and 15 days^3^. Importantly, morbidity and mortality from influenza infections are increased in at-risk patients, such as the elderly and immunocompromised^4,5^. While seasonal influenza epidemics cause a high global disease burden and are important public health concerns, the threat is significantly increased during influenza pandemics. The most recent example is the 2009 H1N1 swine influenza pandemic which infected an estimated 24% of the global population^6^. Another past influenza pandemic occurred in 1968, when the H3 influenza subtype jumped from birds into humans^7,8^. This subtype has continued to circulate in humans, causing seasonal epidemics each year.

The substantial diversity of influenza viruses presents a significant challenge in the development of an effective vaccine. The World Health Organization (WHO) and the Centers for Disease Control (CDC) use influenza virus surveillance to predict the future virus strains of the upcoming influenza season and recommend the annual quadrivalent vaccine formulation that contains a H1N1, H3N2, and an influenza B strain from both the Victoria-like and Yamagata-like lineages. Yearly vaccine effectiveness (VE) is highly variable (10–60%)^9^ and provides limited cross-protective immunity against mismatched strains^10^. Importantly, a meta-analysis examining the seasonal influenza VE from 2004 to 2015 found that vaccine efficacy against the H3N2 subtype was low, even in the case of an antigenic match. Although influenza vaccination provided substantial protection against H1N1 and type B viruses, with pooled VE ranging from 54 to 67%, the pooled VE against H3N2 was only 33% for antigenically matched viruses and 23% for mismatched viruses^11^. Additionally, H3N2-dominated influenza seasons are associated with higher morbidity and mortality as compared to H1N1 or influenza B seasons^12,13^. The poor vaccine efficacy against H3N2 viruses and increased disease burden highlight a crucial need for improved vaccine technologies which generate more cross-reactive immunity against divergent H3N2 influenza viruses.

The Epigraph vaccine antigen designer uses a graph-based algorithm to create a cocktail of vaccine antigens that are designed to maximize the potential epitope coverage of a highly diverse population^14^. The Epigraph algorithm has been used to predict therapeutic HIV vaccine candidates^15^ and was evaluated in vivo as a Pan-Filovirus vaccine^16^. We previously demonstrated the efficacy of the Epigraph vaccine immunogen design strategy as a universal swine H3 (swH3) influenza vaccine^17^. Vaccination of mice and swine with the Epigraph swH3 vaccine induced superior levels of cross-reactive antibodies and T-cell responses as compared to the commercial whole-inactivated quadrivalent swine influenza vaccine, FluSure. Here, we explore Epigraph immunogen design for the development of a universal human H3 influenza vaccine. A cocktail of three H3 hemagglutinins (HA), a surface glycoprotein of influenza, were computationally designed using the Epigraph vaccine designer and expressed in a replication-defective Adenovirus type 5 vector (HAdV-5). We evaluated Epigraph vaccine-induced immunity and protection in both mice and ferret animal models and demonstrate increased cross-reactivity and protection against diverse human H3N2 viruses isolated between 1968 and 2019 compared to a traditional egg-based, inactivated quadrivalent influenza vaccine, FluZone.

## Results

### Development and characterization of the H3 epigraph HA vaccine

The human H3 Epigraph immunogens were designed with the Epigraph vaccine designer tool using all 5709 unique H3 HA amino-acid sequences available on the Influenza Research Database as of 15 November 2017. The isolation years of these sequences range from 1968 to 2017. The Epigraph tool is a graph-based algorithm that creates a cocktail of immunogens designed to maximize potential epitope coverage from a population of viral sequences^14,15^. The Epigraph algorithm calculates the frequency of each 9-mer sequence in the target population and uses a graph-based algorithm to trace a path across the HA protein that contains the most common potential 9-mer epitopes in the target virus population^17^. This results in a full-length HA protein with the most common epitopes in the population which, by design, is centrally located on the phylogenetic tree (Epigraph 1). The epitopes identified in Epigraph 1 are excluded and the algorithm traces a path containing the second most common 9-mer epitopes, resulting in Epigraph 2. Similarly, Epigraph 3 consists of the third most common epitopes in the population. The resulting Epigraph immunogens represent a trivalent cocktail that is computationally designed to maximize the coverage of potential epitopes in the viral population along with minimizing rare epitopes which might result in strain-specific immunity.

**Fig. 1:**
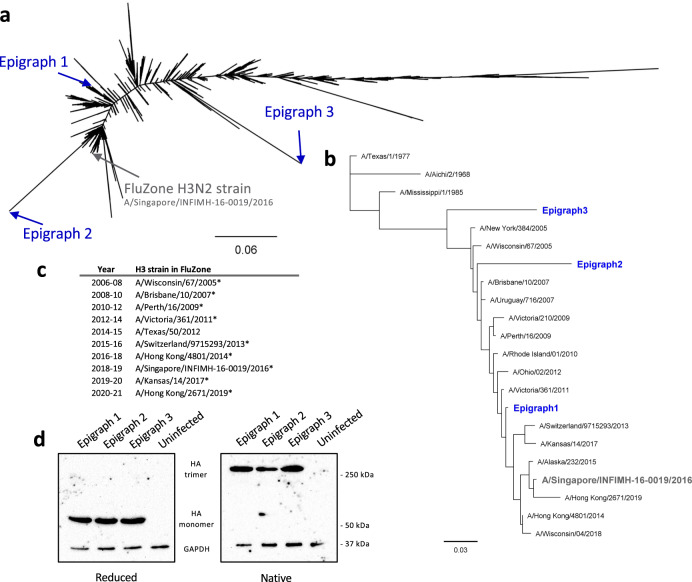
Characterization of the H3 Epigraph vaccine constructs. **a** The Epigraph vaccine designer tool was used to create a cocktail of three H3 Epigraph immunogens (Epigraph 1, 2, and 3), which are computationally designed to maximize potential epitope coverage in the total H3 population. These Epigraph H3 immunogens were aligned back to the 5709 unique H3 HA sequences using ClustalW and a maximum likelihood phylogenetic tree was created to visualize the relationship between the vaccine immunogens and the H3 population. **b** A panel of 18 H3 strains was selected to examine the cross-reactivity immunity after vaccination. The selected strains span the H3 phylogenic tree and include both contemporary (2005–2019) and historical (1968–1985) strains. A maximum-likelihood tree was constructed using PhyML to visualize the relationship between the assay strains, the Epigraph vaccine immunogens (blue), and the FluZone strain (gray; A/Singapore/INFIMH-16-0019/2016). **c** A table of H3 strains recommended for the FluZone vaccine each year since 2006. An asterisk indicates inclusion in this study. **d** The three H3 Epigraph immunogens were cloned into replication-defective Adenovirus type 5 (HAdV-5) vectors and the HA protein expression was confirmed by western blot. Reducing conditions were used to observe HA monomers while native conditions were to obverse HA trimers. GAPDH is used as a cellular protein loading control.

The resulting three Epigraph immunogens localize across a maximum likelihood phylogenetic tree constructed using the entire population of unique H3 HA protein sequences (Fig. 1a). A BLAST search found that the most similar wild-type sequences are as follows: Epigraph 1- A/Ishikawa/90-205v/2013 (99.65% sequence homology), Epigraph 2- A/Japan/4426/2015 (92.58% sequence homology), Epigraph 3- A/Denmark/143/2005 (91.34% sequence homology). In the interest of improving current vaccine strategies, we compared our Epigraph vaccine to the commercial inactivated vaccine, FluZone 2018–19 formulation, which is a quadrivalent formulation containing the H3N2 strain A/Singapore/INFIMH-16-0019/2016. In order to evaluate the cross-reactivity of our vaccine, we selected a panel of 18 representative H3 viruses that represent 15 modern contemporary strains (2005–2019) and three historical strains (1968–1985). A maximum-likelihood tree was constructed to evaluate the relationship of the three Epigraph immunogens to this panel of representative viruses (Fig. 1b; Supp. Table 1). Importantly, this panel represents much of the diversity of the H3 population and contains both contemporary and historical strains. This panel also contains all but one of the H3N2 strains recommended for use in the seasonal influenza vaccines for the past 15 years (Fig. 1c), thereby representing much of the diversity of recent strains circulating in the human population. In addition, this panel contains two viruses isolated after our Epigraph immunogens were designed in order to evaluate if any potential protection against future emergent strains can be detected.

The three H3 Epigraph immunogens were cloned into the replication-defective viral-vector HAdV-5 individually and HA protein detection was confirmed by Western blot (Fig. 1d). HA expression was confirmed in both reducing and native conditions to examine HA monomers and potential trimer formation, respectively. The development of trimers after expression of the Epigraph HA immunogens would indicate that, although these immunogens are synthetic in nature, each Epigraph immunogen has the potential to structurally resemble natural HA proteins. Proper HA trimerization could enable the induction of both conformational antibody responses in addition to linear antibody and T-cell responses.

### Epigraph vaccination induces highly cross-reactive antibody responses in mice

**Fig. 2:**
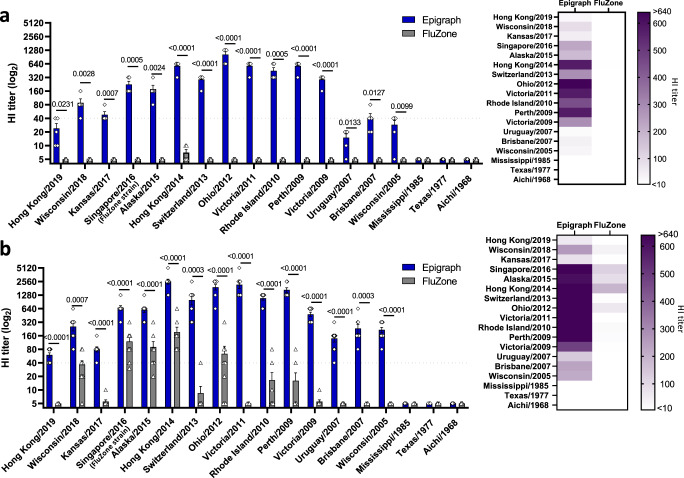
Epigraph immunization induces cross-reactive antibody responses in mice against a panel of 18 representative H3 viruses. BALB/c mice were vaccinated with 10^10^ vp of HAdV-5-Epigraph and compared to FluZone-vaccinated mice. Antibody responses were measured using a HI assay against the 18 H3 representative strains for both a single immunization (**a**) or after boosting (**b**). A heat map of these HI titers was constructed to further visualize the total cross-reactive antibody response of each vaccine. Data are presented as the mean with standard error (SEM) (single immunization *n* = 5; prime boost *n* = 10; unpaired *T* test).

To evaluate the cross-reactive antibody response, 10 BALB/c mice per group were vaccinated with 10^10^ virus particles (vp) of the HAdV-5-Epigraph vaccine, which consisted of equal ratios of the three HAdV-5-Epigraphs (3.33 × 10^9^ vp each) delivered as a cocktail. The Epigraph vaccine was compared to mice vaccinated with either FluZone, the commercial inactivated vaccine, or a PBS sham vaccine. Antibody responses were evaluated three weeks after a single immunization (Fig. 2a) or two weeks after boosting (Fig. 2b) using a hemagglutination inhibition (HI) assay against the panel of 18 representative H3 viruses. In humans, HI titers of at least 1:40 are generally accepted as corresponding to a 50% reduction in the risk of influenza infection and are used as a “threshold“ titer for predicted protection^18–20^. Vaccination with the Epigraph immunogens resulted in strong cross-reactive antibody response, with HI titers ≥40 to 12 of the 18 (67%) H3 strains after a single immunization, while a single immunization of FluZone did not result in significant development of antibodies against any strains. After boosting, Epigraph-vaccinated mice showed HI titers ≥40 to 15 of the 18 (83%) H3 strains. In contrast, FluZone boosted mice showed a more strain-specific response, with HI titers ≥40 to only 4 of the 18 (22%) H3 strains. FluZone showed protective antibody titers to the matched FluZone strain and other similarly related strains, with only limited cross-reactivity with mismatched viruses. Importantly, Epigraph vaccination-induced protective HI titers to 100% of the contemporary H3 strains after boosting, with the absence of HI antibody titers to only the three highly divergent historical H3 strains. The substantial differences in HI titers between the Epigraph and FluZone vaccines are clearly demonstrated by the heat maps (Fig. 2). Most notably, Epigraph vaccination resulted in protective antibody titers to Hong Kong/2019 and Wisconsin/2018, which were isolated after the computational design of the Epigraph immunogens, indicating a significant breadth of protection.

### Epigraph vaccination induces cross-reactive T-cell responses against both contemporary and historical H3 strains

**Fig. 3:**
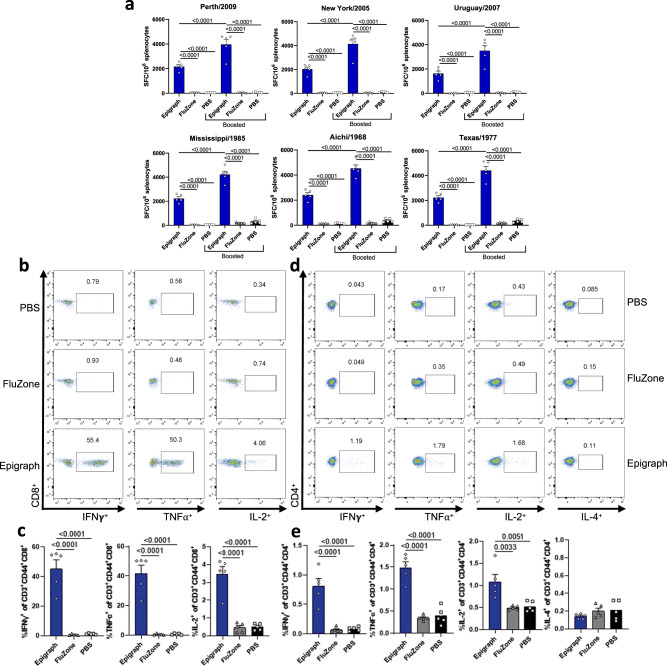
Epigraph immunization induces cross-reactive T-cell responses in mice against 6 diverse H3 viruses. **a** The total T-cell response after vaccination of BALB/c mice (*n* = 5) was determined using an interferon IFN-γ ELISpot with peptide libraries for six H3 strains. T-cell responses are reported as spot-forming cells (SFC) per million splenocytes (*n* = 5; one-way ANOVA with Tukey multiple comparisons). Cytokine profiles were determined using ICS and flow cytometry on splenocytes from vaccinated BALB/c mice (*n* = 5) harvested 2 weeks after boosting and stimulated ex vivo with pooled Perth/2009 peptides (**b**–**e**). Splenocytes were gated on CD3^+^, CD44^high^, and then CD8^+^ (**b**, **c**) or CD4^+^ (**d**, **e**) to determine HA-specific T_EM_ cells. Representative FACS plots for cytokine production for CD8^+^ (**b**) and CD4^+^ (**d**). Levels of cytokine-producing CD8^+^ (**c**) and CD4^+^ (**e**) (*n* = 5; one-way ANOVA with Tukey multiple comparisons). Data are expressed as the mean with standard error (SEM).

Next, we evaluated the ability of these Epigraph immunogens to induce cross-reactive T-cell responses, since T cells have been shown to play an important role in viral clearance during influenza virus infection^21,22^. We evaluated T-cell responses using an IFN-γ ELISPOT and peptide libraries representing three contemporary and three historical strains. Epigraph vaccination-induced highly significant cross-reactive T-cell responses against all six strains after only a single immunization and this response increased (~two-fold) upon boosting (Fig. 3). In contrast, the inactivated virus vaccine FluZone did not induce significant T-cell responses against any of the strains. Importantly, Epigraph vaccination-induced significant and robust T-cell responses to the three historical H3 strains despite not inducing detectable antibody responses as measured by HI titers.

To evaluate the cytokine profile and development of T effector memory (T_EM_) cells, we performed intracellular cytokine staining (ICS) and flow cytometry on splenocytes from boosted mice stimulated ex vivo with pooled peptides from the Perth/2009 HA protein. In order to determine which subsets of T cells were contributing to the overall vaccine-induced immunity, we examined ICS for Th1-associated cytokines interferon-γ (IFN-γ), tumor necrosis factor-α (TNF-α), and interleukin-2 (IL-2) and the Th2-associate cytokine interleukin-4 (IL-4). Representative scatter plots of the CD8+ cells and CD4+ cells induced by the vaccines are shown (Fig. 3b, d, respectively). The sum total responses induced by the vaccines in CD8+ and CD4+ T cells are shown (Fig. 3c, e, respectively). Clearly, the Epigraph vaccine induced a strong Th1-associated immune response while the FluZone group did not induce any significant cellular immunity as compared to the PBS control vaccinated animals. In all cases, the Th1-associated intracellular cytokine expression was significantly greater in the Epigraph group than in the Fluzone group. The Th2-associated IL-4 ICS did not reveal any significant differences between the vaccinated groups and were statistically insignificant as compared to the negative control PBS group. The gating strategy is described (Supp. Figure 1).

### Epigraph vaccination protects mice from contemporary and historical influenza virus challenges

**Fig. 4:**
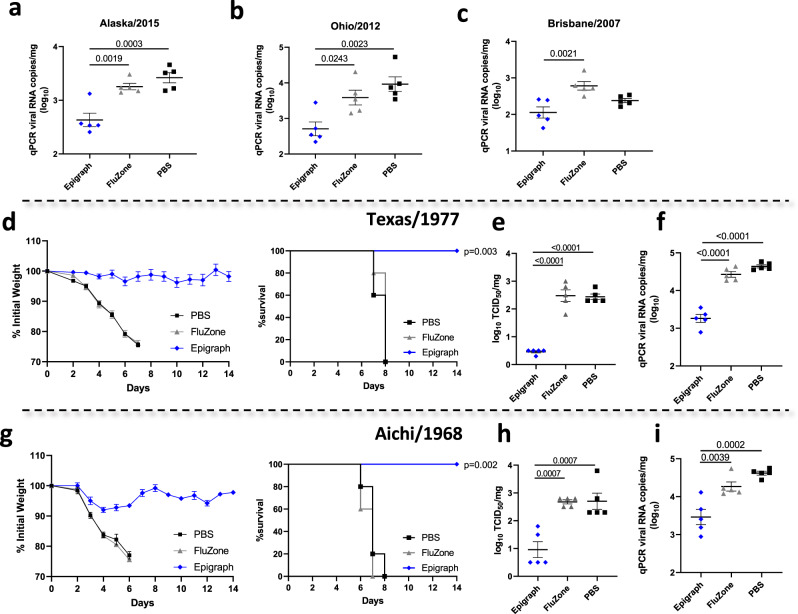
Protection against challenge with contemporary and historical H3 viruses in mice. BALB/c mice were vaccinated with 10^10^ vp of HAdV-5-Epigraph, the FluZone vaccine, or a mock PBS vaccine. Mice were challenged intranasally three weeks after immunization with 10^3.6–3.8^ TCID_50_ of contemporary strains Alaska/2015 (**a**), Ohio/2012 (**b**), or Brisbane/2007 (**c**) and sacrificed 3 days post challenge to examine lung viral titers (*n* = 5; one-way ANOVA with Tukey multiple comparisons). For lethal challenge with historical H3 strains, BALB/c mice (*n* = 10) were challenged intranasally 3 weeks post immunization with 10 MLD_50_ of Texas/1977 (**d**–**f**) or Aichi/1968 (**g**–**i**). Mice were monitored for weight loss over 14 days and animals that showed 25% weight loss were humanely euthanized (*n* = 5; Kaplan–Meier log-rank test compared to the sham vaccinated group). Weight loss data are censored after the first animal death in a group to prevent survival bias. Three days post infection, five mice per group were sacrificed to examine lung viral titers by TCID_50_ (**e**, **h**) and qPCR (**f**, **i**) (*n* = 5; one-way ANOVA with Tukey multiple comparison). Data are presented as the mean with standard error (SEM).

To determine if the cross-reactive immunity induced by Epigraph vaccination protected mice from influenza infection, we challenged vaccinated mice with both contemporary and historical H3 influenza strains three weeks after a single immunization. Unfortunately, contemporary H3 strains, unlike historical strains, are not very pathogenic in mice and pathogenicity did not increase after our attempt to mouse-adapt four H3 strains by 13 serial passages in mice lungs. Therefore, to examine protection against contemporary strains in a mouse model, we examined lung viral titers three days after challenge using quantitative Polymerase chain reaction (qPCR) (Fig. 4a–c). Epigraph vaccination significantly reduced lung viral titers as compared to FluZone-vaccinated animals after challenge with Alaska/2015, Ohio/2012, and Brisbane/2007 contemporary strains, supporting the efficacy of the Epigraph vaccine in protection against contemporary strains.

We then evaluated the ability of Epigraph vaccination to protect against lethal challenge with 10 mouse-lethal dose 50% (MLD_50_) of two historical H3 strains (Fig. 4d–i). Mice were monitored for weight loss and humanely sacrificed if they reached 25% weight loss. In addition, groups of mice were sacrificed on day 3 post challenge in order to examine lung viral titers by both tissue-culture infectious dose 50% (TCID_50_) and qPCR to evaluate infectious virus and viral RNA copies, respectively (Fig. 4e, f, h, i). A single Epigraph immunization completely protected mice from weight loss and death after challenge with Texas/1977 (Fig. 4d). In contrast, FluZone and PBS sham vaccinated animals all succumbed to challenge by day 8. In addition, Epigraph vaccination significantly reduced lung viral titers as compared to both FluZone and PBS-vaccinated animals (Fig. 4e, f). Similarly, a challenge with another historical strain, Aichi/1968, resulted in all FluZone and PBS-vaccinated animals succumbing to disease by day 8 while Epigraph-vaccinated animals were completely protected from death and only experienced moderate weight loss (~8% weight loss) before recovering (Fig. 4g). Again, Epigraph immunization significantly reduced day 3 lung viral titers after challenge as compared to FluZone and PBS sham vaccinated animals (Fig. 4h, i). This demonstrates that Epigraph vaccination still results in complete protection from death after challenge with historical H3 strains, despite inducing only cross-reactive T-cell responses against the historical H3 strains and not inducing cross-reactive antibodies as measured by HI assay.

### Cross-reactive T cells contribute towards protection after challenge with historical strains

**Fig. 5:**
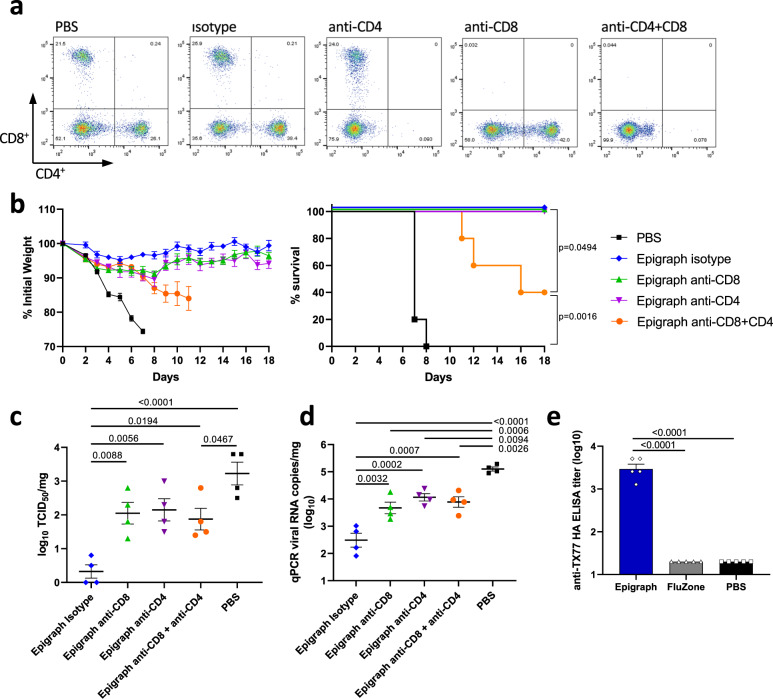
T-cell depletion during lethal Texas/1977 virus challenge reduces Epigraph vaccine efficacy. BALB/c mice were vaccinated (*n* = 9) with 10^10^ vp of HAdV-5-Epigraph and depleted for T cells through administration of 200 μg of anti-mouse CD8, anti-mouse CD4, both anti-CD4 and CD8, or isotype control on day −3, −1, and 1 of challenge. **a** All mice were bled on day 1 post challenge via submandibular vein to confirm >98% T-cell depletion by flow cytometry. **b** Mice were challenged intranasally with 10 MLD_50_ of Texas/1977 and monitored for weight loss over 18 days. Animals that showed 25% weight loss were humanely euthanized (*n* = 5; Kaplan–Meier log-rank test). Weight loss data are censored after the first animal death in a group to prevent survival bias. Three days post infection, four mice per group were sacrificed to examine lung viral titers by TCID_50_ (**c**) and qPCR (**d**) (*n* = 4; one-way ANOVA with Tukey multiple comparison). **e** An ELISA was performed to examine the presence of binding, non-neutralizing antibodies against Texas/1977 after a single immunization (*n* = 5; one-way ANOVA with Tukey multiple comparison). Data are presented as the mean with standard error (SEM).

To further examine the contribution of cross-reactive T cells in the protection observed after challenge with historical H3 strains, we performed a T-cell depletion study. Mice were vaccinated and challenged with Texas/1977 as described above but were administered 200 μg of anti-CD8, anti-CD4, both anti-CD8 and anti-CD4, or isotype control antibody on day −3, −1, and 1 of the challenge to deplete the respective T-cell population. All mice were bled on day 1 and confirmed to have greater than 98% T-cell depletion using flow cytometry (Fig. 5a). Epigraph-vaccinated mice treated with an isotype control were completely protected from weight loss and death whereas the PBS-vaccinated mice quickly succumbed to lethal challenge (Fig. 5b). Epigraph-vaccinated mice which were single depleted for either CD4 or CD8 showed increased weight loss (~10% loss), however, these mice began to recover after day 8 post challenge. In contrast, the double-depleted mice continued to lose weight and three of the five animals succumbed to lethal infection. In addition, Epigraph-vaccinated mice with either single or double T-cell depletion showed a significant increase in lung viral titers as compared to non-depleted Epigraph-vaccinated animals (Fig. 5c, d). Epigraph-vaccinated animals with intact T-cell immunity showed the largest reduction in lung viral titers. Lastly, an ELISA showed that Epigraph vaccination does induce non-neutralizing anti-Texas/1977 binding antibodies which are not detected by HI assay (Fig. 5e). These results demonstrate that cross-reactive T cells induced by the Epigraph vaccine significantly contribute to protection from challenges with a lethal historical H3 strain.

### Individual Epigraph immunogens contribute differently to cross-reactive antibodies and T-cell responses in mice

**Fig. 6:**
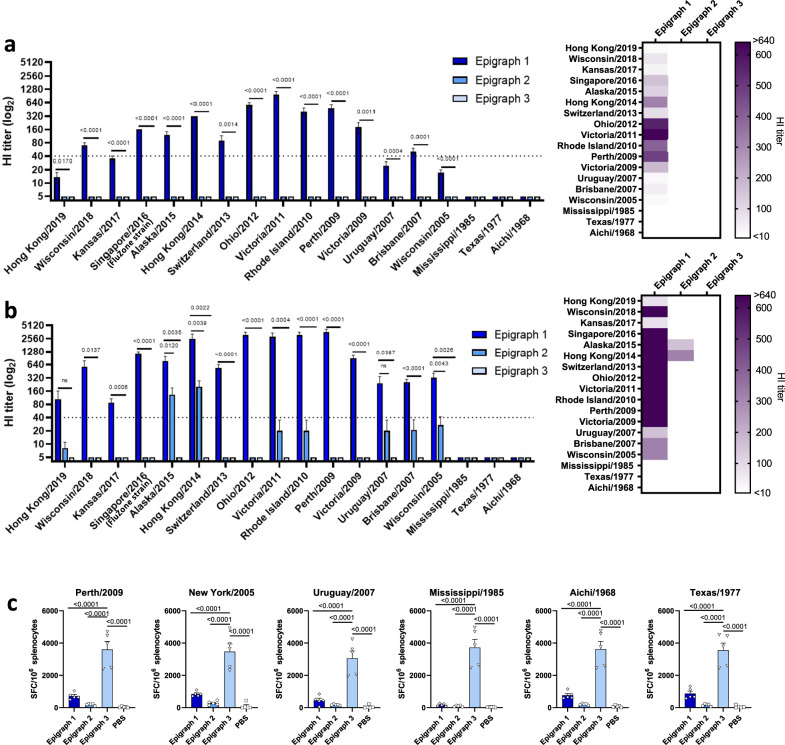
Contribution of each individual Epigraph immunogen towards the cross-reactive antibody and T-cell responses. To examine the contribution of each individual Epigraph immunogen, BALB/c mice (*n* = 5) were immunized with 3.3 × 10^9^ vp of HAdV-5-Epigraph 1, 2, or 3 individually. Antibody responses were measured using a HI assay against the 18 H3 representative strains for both a single immunization (**a**) or after boosting (**b**). A heat map of these HI titers was constructed to further visualize the total cross-reactive antibody response of each vaccine. **c** The total T-cell response after boosting was determined using an interferon IFN-γ ELISpot with peptide libraries for six H3 strains. T-cell responses are reported as spot-forming cells (SFC) per million splenocytes (*n* = 5; one-way ANOVA with Tukey multiple comparisons). Data are presented as the mean with standard error (SEM).

Although the H3 Epigraph immunogens were designed to be delivered as a trivalent cocktail, we wanted to determine the contribution of each individual Epigraph immunogen. BALB/c mice were vaccinated with individual Epigraph immunogens of either Epigraph 1, 2, or 3, and cross-reactive antibody and T-cell responses were evaluated. After a single immunization, only the Epigraph 1 immunogen vaccine-induced cross-reactive HI antibody responses (Fig. 6a). After boosting, the Epigraph 1 immunogen vaccine induced the most cross-reactive antibody responses, as compared to the Epigraph 2 and 3 immunogen vaccines. However, after boosting, the Epigraph 2 immunogen vaccine now showed cross-reactive antibodies with some H3 virus strains in the panel (Fig. 6b). We next examined T-cell responses after vaccination and found that immunization with the Epigraph 3 immunogen vaccine induced the strongest cross-reactive T cells against all six H3 strains in our T-cell panel (Fig. 6c). Therefore, in this mouse model, Epigraphs 1 and 2 induced cross-reactive antibodies, while Epigraph 3 induced the most cross-reactive T cells, indicating that a mixture of the trivalent cocktail is optimal for the best broad cross-reactive immunity.

### Epigraph vaccination-induced highly cross-reactive antibody responses in ferrets

**Fig. 7:**
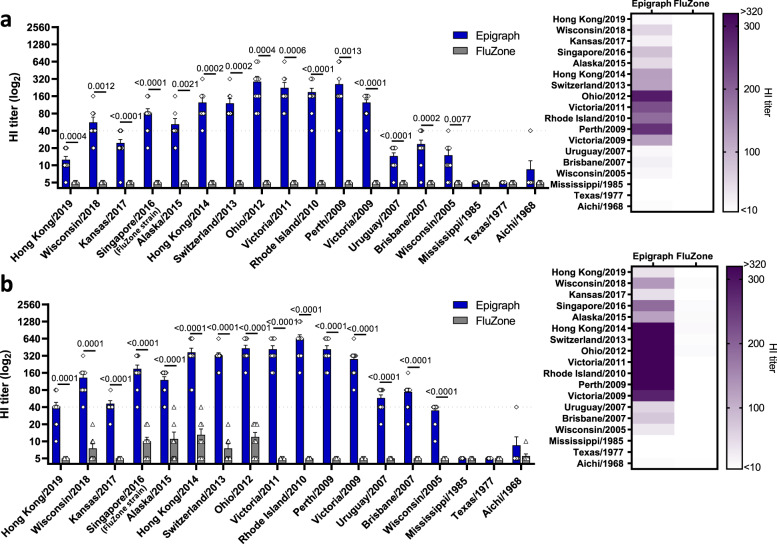
Epigraph immunization induces cross-reactive antibody responses in ferrets against a panel of 18 representative H3 viruses. Ferrets (*n* = 10) were immunized with 5 × 10^10^ vp of the HAdV-5-Epigraph vaccine, a full human dose of FluZone, or a mock PBS vaccine. Antibody responses were measured using a HI assay against the 18 H3 representative strains for both a single immunization (**a**) or after boosting (**b**). A heat map of these HI titers was constructed to further visualize the total cross-reactive antibody response of each vaccine. Data are presented as the mean with standard error (SEM) (*n* = 10; unpaired *T* test).

Our final in vivo analyses were performed using the ferret influenza model. Ferrets are considered the ideal small animal model for human influenza virus infections since they are susceptible to infection, display more human-like influenza symptoms, and the pathology more closely resembles that of a human infection. After a single immunization, the sera from Epigraph immunized ferrets had significantly higher HI titers against 15 out of 18 virus strains as compared to the control commercial vaccine, FluZone (Fig. 7a). In contrast, the FluZone did not induce significant HI titers against any of the virus strains after a single immunization. Upon boosting with a second dose of vaccine, the Epigraph vaccine-induced HI titers ≥40 against 14 out of 15 contemporary virus strains, however, the FluZone vaccine did not induce HI titers ≥40 against any of the virus strains (Fig. 7b). As seen in the mice studies, the ferrets also failed to induce significant HI titers against the historical virus strains.

### Epigraph vaccination reduces influenza disease in ferrets after challenge with two contemporary H3 viruses

**Fig. 8:**
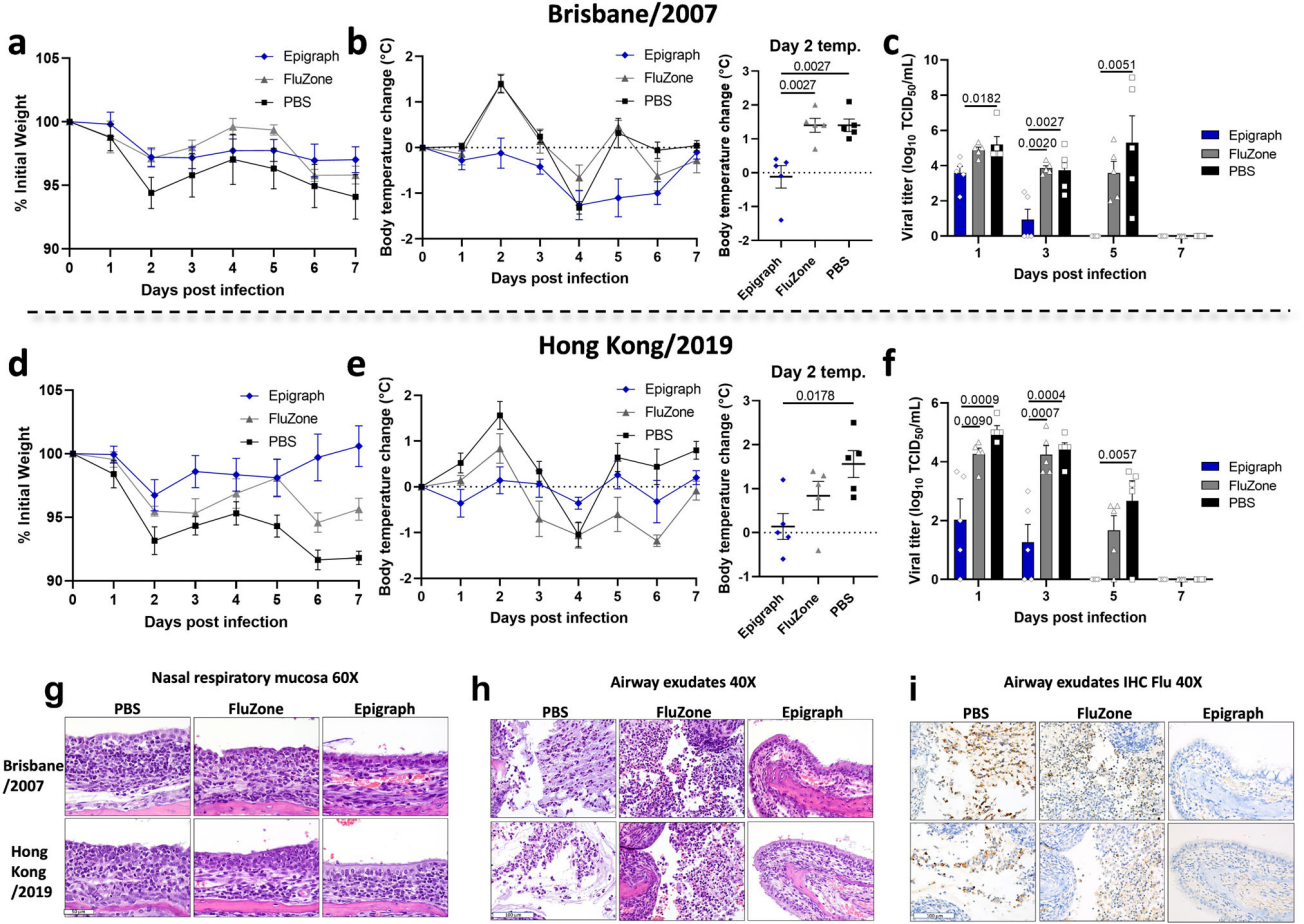
Epigraph immunization protects ferrets against challenge with two contemporary H3 viruses. Ferrets were immunized twice with 5 × 10^10^ vp of HAdV-5-Epigraph, the FluZone vaccine, or a mock PBS vaccine. Two weeks after boosting, ferrets (*n* = 5) were challenged intranasally with 10^5^ TCID_50_ of contemporary strains Brisbane/2007 (**a**–**c**) or Hong Kong/2019 (**d**–**f**). Ferret’s weight (**a**, **d**) and body temperature (**b**, **e**) were monitored over 14 days (day 1–7 *n* = 5; day 8–14 *n* = 3). Peak body temperature at day 2 post infection (*n* = 5; one-way ANOVA with Tukey multiple comparison). On day 1, 3, 5, 7, and 9 post infection, nasal washes were taken to evaluate viral titer kinetics by TCID_50_ (day 1, 3, 4, 7 *n* = 5; day 9 *n* = 3; one-way ANOVA with Tukey multiple comparison). At day 7 post infection, two animals per group were sacrificed to perform immunohistochemistry staining. The influenza pathology for the nasal respiratory mucosa H&E, airway exudates H&E, and airway exudates influenza virus immunohistochemistry are shown (**g**–**i**, respectively. Data are presented as the mean with standard error (SEM).

At lastly, we challenged the immunized ferrets with two human influenza virus strains. One virus strain was included in the design of the Epigraph vaccine (Brisbane/2007) and a second strain represents a strain that was isolated after the Epigraph design (Hong Kong/2019). While weight loss after virus challenge was modest (<10% initial weight lost), overall the Epigraph immunized ferrets showed the least weight loss as compared to the FluZone and PBS control groups (Fig. 8a, d). More noticeably, the Epigraph immunized ferrets showed no significant fever after the challenge while the FluZone and PBS control groups did have significantly higher temperatures, especially on day 2 post challenge (Fig. 8b, e). Importantly, the Epigraph immunized ferrets had significantly lower nasal virus titers as compared to the FluZone and PBS control groups after both viral challenges (Fig. 8c, f). In fact, the nasal titers were nearly undetectable by day 3 and completely undetectable by day 5. However, the FluZone and PBS immunized ferrets had significant viral titers at days 3 and 5 post challenge.

### Epigraph vaccination reduced visible inflammatory response in ferret lung histopathology

Although the Brisbane and Hong Kong virus strains produced similar lesions in the nasal mucosa of each vaccination group, the type, and severity of lesions differed markedly between PBS-, FluZone-, and Epigraph-vaccinated ferrets (Fig. 8g). In PBS-vaccinated ferrets, the submucosa and mucosal epithelium were diffusely infiltrated by inflammatory cells, which consisted primarily of lymphocytes and macrophages, accompanied by lesser numbers of granulocytes. The surface epithelium was disrupted and thinned due to loss of respiratory epithelial cells and inflammatory cell infiltrates. The remaining epithelial cells were round to cuboidal in shape, and lacked cilia. In the FluZone-vaccinated ferrets, inflammatory cell infiltrates were also mixed, but included abundant eosinophils, which were the predominant cell type within the surface epithelial layer and airway exudates. Disruption of the surface epithelium was less severe than in PBS-vaccinated ferrets, but respiratory epithelia cells were also round to cuboidal and lacked cilia. In marked contrast, inflammatory cells in the Epigraph-vaccinated ferrets were milder and mostly restricted to the submucosa. Respiratory cells were generally columnar and ciliated. The composition of airway exudates also differed markedly between PBS-, FluZone-, and Epigraph-vaccinated ferrets (Fig. 8h). In PBS-vaccinated ferrets, these exudates consisted primarily of sloughed epithelial cells, along with some granulocytes. In comparison, the exudates in FluZone-vaccinated ferrets were more densely cellular and consisted mostly of eosinophils. Cellular exudates were not present in the Epigraph-vaccinated ferrets. Immunohistochemical staining for viral antigen (Fig. 8i) showed intense labeling of numerous sloughed epithelial cells in PBS-vaccinated ferrets. The labeling was less intense in the FluZone-vaccinated ferrets and was most concentrated within the smaller granulocytes (eosinophils). Virus antigen was not detected by immunohistochemistry in the Epigraph-vaccinated ferrets.

## Discussion

The low VE against the H3 subtype^11^ and increased morbidity and mortality associated with H3N2-dominated influenza seasons^12,13^ highlight the crucial need for improved H3N2 influenza vaccines. Therefore, we utilized the Epigraph vaccine design algorithm to design broadly cross-reactive H3 HA. Our goal was to maximize the potential of inducing strong cross-reactive immune responses to assess the platform’s potential to create a universal H3 vaccine. We have previously demonstrated the efficacy of Epigraph HA immunogens in the creation of a universal swine H3 vaccine^17^ and here we show similar improved cross-reactive efficacy when applied to a human H3 influenza virus vaccine. Mice immunized with the Epigraph vaccine developed highly cross-reactive HI titers and T-cell responses against a large panel of diverse H3 strains and better-protected mice from challenges with both contemporary and historical H3 strains as compared to the commercial inactivated vaccine FluZone.

HI titers are the current benchmark for evaluating the efficacy of influenza vaccines, with an HI titer of at least 1:40 generally accepted as corresponding to a 50% reduction in the risk of influenza infection in humans and is often used as a ‘threshold’ protective titer^18–20^. In our study, mice immunized with our Epigraph vaccine-induced protective HI titers (≥40) to 83% of the highly diverse H3 viruses in our panel after boosting, whereas two FluZone immunizations induced a more strain-specific response, with protective HI titers (≥40) to only 22% of the viruses. Most importantly, two immunizations with the Epigraph vaccine results in protective HI titers to 100% of contemporary strains, two of which were isolated *after* the Epigraph immunogens were designed. This indicates that HAdV-5-vectored Epigraph vaccination induces highly cross-reactive immunity that can continue to provide serological protection against divergent H3 strains. It should be noted that the viruses used for our HI titers were grown in embryonated eggs, which may not fully translate to a response that would be seen against circulating H3N2 strains. Furthermore, Epigraph vaccination-induced protective HI titers against all H3N2 strains recommended for use in seasonal influenza vaccines for the past 15 years, with the exception of one strain that was not available in our panel. These promising results support that the Epigraph vaccine is capable of protecting against 15 years of viral H3 diversity circulating in humans and can continue to protect against emerging H3 (2018 and 2019) strains that were not included in Epigraph immunogen design.

The results that were observed in mice were corroborated with our ferret studies. Ferrets are considered the ideal small animal model for influenza vaccine studies^23^ using human influenza viruses for several reasons. They are more susceptible to human influenza viruses than mice, exhibit a disease pathology and symptoms that are very similar to human infections, and are outbred. This is especially important because this eliminates potential MHC-class biases that may occur in an inbred mouse model. Again, after boosting with the Epigraph vaccine the ferrets had HI titers ≥40 against 14 of 15 (93%) of contemporary virus strains tested, including two strains that did not exist when the Epigraph immunogens were designed. What was especially noteworthy was two doses of FluZone in ferrets failed to induce any HI titers ≥40, even against the matched strain. This signifies an alarming need for improvement on current influenza vaccines employed. The results were further confirmed in the ferret influenza virus challenge studies. Vaccination with our Epigraph vaccine resulted in the least overall weight loss, protection from fever and significantly reduced nasal viral titers against both Brisbane/2007 (genetic group a) and Hong Kong/2019 (genetic group 3 C.2a1b + T135K-B) influenza virus strains. Pathology results also confirmed that the Epigraph-vaccinated ferrets exhibited advanced levels of protection as compared to the PBS and FluZone-vaccinated groups. Again, these data confirm that the Epigraph vaccine was capable of inducing protection against past and future influenza virus strains.

Epigraph vaccination not only induces a broadly cross-reactive antibody response, but the induced antibody titers are much stronger when compared to the commercial influenza vaccine, FluZone. While an HI titer of 1:40 is accepted as the threshold antibody titer that results in a 50% reduction in the risk of influenza infection, the stronger the HI titer, the greater the level of protection. In addition, higher titers are often required to protect at-risk populations, such as pediatric or elderly populations^19,24^. Studies have estimated that HI titers ≥160 correlate with approximately 90% reduction in the risk of influenza infection^18,25,26^. Epigraph vaccination showed a robust antibody response, with HI titers ≥160 to 12 of the 15 contemporary H3 strains after boosting and significantly higher antibody responses as compared to FluZone for all contemporary H3 viruses in mice with similar results observed in ferrets. Interestingly, the Epigraph vaccine induced more than a 5-fold stronger antibody response against Singapore/2016 (genetic group 3 C.2a1), the H3 strain incorporated in FluZone. It is generally considered that a matched vaccine would be best suited against a homologous challenge. Here our data indicate otherwise and this has been observed in our previous studies where, even the cellular immunity was greater in the Epigraph-vaccinated animal as compared to the homologous vaccine^17^. These data indicate that HAdV-5-Epigraph vaccination not only induces highly cross-reactive antibodies, but these antibodies are observed at high titers. Moreover, the increased levels of immunity induced by the Epigraph vaccine may correlate with durability where the higher levels of immunity are maintained for a much longer period of time, as compared to the FluZone induced vaccine responses that very rarely reach beyond a 1:160 HI titer and are known to wane relatively quickly post-vaccination^27^. While our application of the Epigraph design algorithm attempted to enrich for T-cell epitopes for inclusion in our vaccine, this would not preclude B-cell epitopes from being included as well. This is especially true when considering how the algorithm works to construct the immunogen. There is a limited number of residues in the protein, meaning T-cell and B-cell epitopes are likely to overlap. Further mapping of B-cell epitopes would need to be done to identify the important regions for antibody production. Additionally, it is possible the stronger T-cell response to the HAdV-5-Epigraph vaccine may lead to additional interaction between T- and B-cells which could lead to increased production of antibody to prevent viral infection. Further work to explore this phenomenon is planned.

T cells have been shown to play an important role in protection from influenza virus infection in humans, both in facilitating viral clearance^21,22^ and contributing to long-lasting immunity^28–31^. Immunization of mice with the Epigraph vaccine-induced highly cross-reactive T-cell responses, with robust responses against both contemporary and historical strains. Importantly, although Epigraph vaccination did not induce significant HI antibody responses to historical H3 strains, vaccination-induced highly cross-reactive T-cell responses against all three historical virus strains contained in our panel and afforded complete protection against mortality after challenge with two lethal historical viruses. While non-HI antibodies may also play a role in this protection, the absence of T cells significantly altered the protection of mice from lethal challenge. In our study, cross-reactive T-cell responses induced after Epigraph vaccination were found to significantly contribute to protection against lethal historical H3 challenge, with depletion of CD4 and CD8 T cells resulting in extreme weight loss, increased death, and higher lung viral titers as compared to non-depleted Epigraph-vaccinated mice. Future T-cell analysis using more contemporary H3 HA peptide libraries, such as that of Hong Kong/2019 or Singapore/2016, would further strengthen our data to show that the cellular immune response is more broadly cross-reactive than is already shown. It is also known that several mutations were gained in the circulating H3N2 influenza viruses after 2002. These mutations occurred throughout the viral genome and included changes in the major antigenic sites of the HA. These changes may delineate between historic and contemporary influenza strains and could account for the differences in Epigraph-induced humoral immunity^32^.

The H3N2 subtype first jumped from birds into humans in 1968 and has continued to evolve in humans, resulting in altered receptor binding preferences^33–35^ and mutations in glycosylation sites^36,37^. As a result, low pathogenicity is observed in mice after infection with contemporary H3N2 virus. Therefore, we used lung viral titers to determine the protection against contemporary strains and found that Epigraph vaccination resulted in significantly reduced lung viral titers as compared to FluZone for three different contemporary H3N2 strains. These data support the efficacy of Epigraph in protecting against H3N2 challenge, both with contemporary and historical H3N2 strains.

The strong immune responses observed against contemporary strains do serve as support the Epigraph algorithm design. Since many more sequences are available from recent years, it is more likely that recent epitopes are incorporated into the design. This may add to the promise of the vaccine design in that the response to vaccination would likely last longer due to more response to circulating strains. It is possible that in the future, antigenic drift or an antigenic shift of the H3 protein in circulating strains could accumulate mutations which may prevent antibody binding of Epigraph-vaccinated sera. If this were to happen, we could design an additional immunogen to incorporate these new sequences into a new vaccine formulation to maintain strong antibody and T-cell responses.

Interestingly, although Epigraph 1 and 2 contributed primarily to cross-reactive antibody responses, Epigraph 3 induced the strongest cross-reactive total T-cell responses in mice. This indicates that each human H3 Epigraph immunogen contributes differently towards the cross-reactive immunity induced by the trivalent cocktail. It is possible that the Epigraph 3 immunogen contains an immunodominant T-cell epitope that is not contained in either of the other two immunogens. If this epitope is not included in Epigraph 1 or 2, this could explain the much stronger T-cell induction measured in our assay from Epigraph 3. It should be noted that Epigraph 1 still induced a strong total T-cell response but was overshadowed by the strong response to Epigraph 3. Future work can utilize T-cell epitope mapping to identify individual epitopes which may vary between each individual immunogen and lead to the different strengths of responses seen in our study. Additionally, challenge studies conducted by delivery of only individual immunogens into mice prior to infection could highlight the important mechanisms of protection from challenge. These studies would further delineate the robust activation we have reported. Ultimately, our data strongly support that a trivalent cocktail of the Epigraph immunogens is likely optimal for induction of broad cross-reactive immunity.

An important note for this study is that a direct comparison of our Epigraph vaccine and FluZone is not entirely equivalent due to the different types of formulations. Delivering a vaccine using a viral-vector is likely to induce some form of immune response to the vector as well, which may skew some of the data towards a stronger immune response than would be seen with a recombinant protein. However, for the purpose of our study, these preliminary results characterize the usefulness of further research into the H3 Epigraph vaccine. There is little consensus for ways to control for viral-vectored immunogen dosing in an animal model, which makes direct viral-vector to recombinant protein comparisons more challenging. Further, all immune correlate data reported in this study is influenza HA specific, so the immune responses reported are against the immunogen cocktail itself. Future studies will address whether vaccinating with a trivalent cocktail of wild-type HAs would produce similar results to Epigraph vaccination, and whether recombinant protein forms of the H3 Epigraph vaccine would be able to recapitulate the immune responses seen in our study. We believe that we would still see improved cross-reactive immunity compared to viral-vectored wild-type HA vaccines or stronger immune responses than FluZone using a recombinant protein delivery of the H3 Epigraph vaccine due to the nature of the immunogen design. By considering the variation seen in the HA in circulating strains and incorporating that variation into the immunogen, Epigraph should be able to enhance the cross-reactive immune potential independent of the HAdV-5 vector.

Here, we designed a universal human H3 Epigraph vaccine and demonstrated that Epigraph vaccination induced highly cross-reactive antibody and T-cell responses that protected against in vivo influenza virus challenges with contemporary and historical H3 virus strains in mice and against two contemporary virus strains in ferrets. With the Epigraph vaccine inducing protective HI titers to 93–100% of contemporary H3 strains and inducing strong cross-reactive T-cell responses, the Epigraph vaccine should be further explored as a more cross-reactive influenza vaccine. Application of this Epigraph vaccine designer strategy to the H1N1 and influenza B subtypes could lead to a universal multivalent influenza vaccine against all subtypes currently circulating in humans. These data support the continued development of Epigraph immunogens as a universal human H3 influenza vaccine that induces significant cross-reactive immunity.

## Methods and materials

### Ethics statement

Female BALB/c mice ages 6-8 weeks were purchased from Jackson Laboratory. Mice were housed in the Life Sciences Annex building on the University of Nebraska – Lincoln (UNL) campus under the Association for Assessment and Accreditation of Laboratory Animal Care International (AAALAC) guidelines. The protocols were approved by the UNL Institutional Animal Care and Use Committee (IACUC) (Project ID 1717) and the St. Jude Children’s Hospital IACUC (Project ID 428). All animal experiments were carried out according to the provisions of the Animal Welfare Act, PHS Animal Welfare Policy, the principles of the NIH Guide for the Care and Use of Laboratory Animals, and the policies and procedures of UNL and St. Jude Children’s Hospital.

### Influenza Viruses

The following influenza viruses were obtained from the Biodefense and Emerging Infectious Diseases Repository: A/Texas/1/1977 (Texas/1977) [NR-3604], A/Mississippi/1/1985 (Mississippi/1985) [NR-3502], A/Aichi/2/1968 (Aichi/1968) [NR-3483], and A/Brisbane/10/2007 (Brisbane/2007) [NR-12283]. The following influenza viruses were obtained from the International Reagent Resource: A/Wisconsin/04/2018 (Wisconsin/2018) [FR-1653], A/Kansas/14/2017 (Kansas/2017) [FR-1686], A/Singapore/INFIMH-16-0019/2016 (Singapore/2016) [FR-1590], A/Alaska/232/2015 (Alaska/2015) [FR-1540], A/Hong Kong/4801/2014 (Hong Kong/2014) [FR-1452], A/Switzerland/9715293/2013 (Switzerland/2013) [FR-1366], A/Ohio/02/2012 (Ohio/2012) [FR-1143], A/Victoria/361/2011 (Victoria/2011) [FR-1027], A/Rhode Island/01/2010 (Rhode Island/2010) [FR-662], A/Perth/16/2009 (Perth/2009) [FR-370], A/Victoria/210/2009 (Victoria/2009) [FR-643], A/Uruguay/716/2007 (Uruguay/2007) [FR-10], A/Wisconsin/67/2005 (Wisconsin/2005) [FR-397], and A/Hong Kong/2671/2019 (Hong Kong/2019) [FR-1744]. The Texas/1977 and Aichi/1968 influenza viruses were mouse-adapted through serial lung passaging in mice five and nine times, respectively. All influenza viruses were grown in specific pathogen-free embryonated eggs and the chorioallantoic fluid was aliquoted and stored at −80 °C. Virus stocks were quantified based on hemagglutination units (HAU) using 50 µL of 0.5% chicken red blood cells and TCID_50_.

### Design and characterization of the epigraph immunogens

The Epigraph Vaccine Designer from the Los Alamos National Laboratories^14,15^ was used to design the three H3 vaccine immunogens. First, all unique human H3 HA sequences, excluding duplicates, were downloaded from the Influenza Research Database, as of 15 November 2017. This included sequences isolated from 1968 to 2017, and the 5709 unique H3 HA sequences were uploaded to the Epigraph Vaccine Designer and run with the following parameters: epitope length: 9, cocktail size: 3. The resulting three HA Epigraph genes (Epigraph 1, 2, and 3) were added back to the H3 sequence population and aligned using ClustalW. The maximum likelihood phylogenetic tree was created using the RAxML-HPC BlackBox tool with a Jones-Taylor-Thornton substitution model using CIPRES Science Gateway V3.3 on the Extreme Science and Engineering Discovery Environment (XSEDE). The tree was visualized using the Geneious 11.1.5 software. The maximum likelihood phylogenetic tree to compare the assay strains to the vaccine strains was created using PhyML 3.3 with a Dayhoff substitution model on the Geneious 11.1.5 software^38^.

### Construction of the replication-defective adenovirus vectors

The three Epigraph HA genes (immunogens) were codon-optimized for human gene expression and synthesized by GenScript. These Epigraph genes were then cloned into a replication-defective E1/E3 deleted Adenovirus type 5 vector using the Ad-Easy Adenoviral Vector System (Agilent). The Epigraph HA genes were individually cloned into the pShuttle-CMV plasmid and cotransformed with pAd-Easy-1 (HAdV-5 genome) into BJ5183 cells for homologous recombination into the E1 region of the HAdV-5 genome^17,39^. The linearized recombinant pAd-Epigraph plasmid DNA was transfected into 293 cells using the PolyFect Transfection Reagent (Qiagen). Virus was amplified by sequential passages in 293 cells until a final amplification using a Corning 10-cell stack (~6300 cm^2^). The virus was purified by 2 sequential CsCl ultracentrifuge gradients, desalted using Econo-Pac 10DG Desalting Columns (Bio-Rad), and stored at −80 °C in 20 mM Tris, 100 mM NaCl, 1 mM MgCl_2_, 10% glycerol (pH 8.0). Virus particles (vp) were quantitated by OD260. The infectious units per mL were determined using the AdenoX Rapid Titer kit according to the manufacturer’s instructions (Clontech Laboratories).

### Western blot

Western blot was used to confirm HA protein expression from the recombinant HAdV-5 vectors. A six-well of confluent 293 cells were infected with 500 virus particles (vp) per cell, incubated at 37 °C and 5% CO_2_, and cells were harvested 48 h after infection. For reduced conditions, cells were denatured using Laemmli buffer plus 2-mercaptoethanol and boiled at 100 °C for 10 min, while native conditions did not receive 2-mercaptoethanol and were not heated. Samples were then passed through a QIAshredder (Qiagen) before running on a 12.5% SDS-PAGE gel. Protein was transferred to a nitrocellulose membrane and blocked for 30 min with 5% milk in TBST. The membrane was incubated overnight at 4 °C with anti-HA Tag HRP conjugated antibody (NB600-391; Novus Biologicals) at 1:1000 and anti-GAPDH (sc-47724) at 1:1000 in TBST 1% milk. The membrane was washed 3× with TBST and probed with secondary goat anti-mouse HRP conjugated antibody (Millipore Sigma #AP308P) at 1:2000 for 30 min at room temperature (RT). The membrane was washed 3× with TBST and developed with SuperSignal West Pico Chemiluminescent Substrate (Thermo Scientific).

### Mouse vaccination and tissue collection

Female BALB/c mice were vaccinated with 10^10^ vp of HAdV-5-Epigraph (a cocktail of the three Epigraph immunogens (3.3 × 10^9^ vp each) at equal ratios to total 10^10^ vp) or 50 μl (600 ng/mouse of total protein) of Fluzone® Quadrivalent Influenza Vaccine, 2018–2019 Formula (NR-51702) which is ~30× the equivalent human dose. The 2018/19 FluZone formula contains the H3 strain A/Singapore/INFIMH-16-0019/2016 IVR-186 (H3N2) which is a fourth of the vaccine’s total protein. All vaccines were compared to a PBS sham vaccinated control group. Immunizations were diluted in PBS and were injected intramuscularly with a 27-gauge needle into both quadriceps in two 25 µl injections. All mice immunizations and bleeds were performed under isoflurane or ketamine and xylazine-induced anesthesia. To examine the immune correlates after a single immunization, mice were sacrificed 3 weeks post-vaccination for collection of sera and splenocytes. To examine immune correlates after two immunizations, another group of mice was boosted with the homologous vaccine and dose 3 weeks after priming and sacrificed two weeks after boosting. Blood was harvested via cardiac puncture and sera was isolated from whole blood using a BD Microtainer Blood Collection Tube (Becton Dickinson). Splenocytes were isolated by passing the spleen through a 40 μm Nylon cell strainer (BD Labware) and red blood cells were lysed using ACK lysis buffer. Splenocytes were resuspended in cRPMI with 10% FBS and used for ELISpot assays.

### Influenza challenges in mice

BALB/c mice (*n* = 10) were vaccinated intramuscularly with 10^10^ vp of the HAdV-5-Epigraph, 600 ng of Fluzone® Quadrivalent Influenza Vaccine, 2018–2019 Formula (NR-51702), or with a PBS sham vaccine. Three weeks later, mice were challenged intranasally with 10^3.6^ TCID_50_ of Brisbane/2007, 10^3.6^ TCID_50_ of Alaska/2015, or 10^3.8^ TCID_50_ of Ohio/2012 and sacrificed 3 days post challenge to examine lung viral titers. For lethal challenge with historical strains, mice were challenged with 10 MLD_50_ of Texas/1977 or Aichi/1968. On day 3 post challenge, five mice from each group were sacrificed to examine lung viral titers by TCID_50_ and qPCR. Lungs were homogenized in PBS, centrifuged at 21,000 × *g* for 10 min, and the lung supernatant collected. The remaining five mice were monitored for weight loss and were euthanized when they lost 25% of their starting weight. For T-cell depletion, mice we challenged as described above but were administered 200 μg of anti-mouse CD8α (Clone 2.43 Cat# BE0061), anti-mouse CD4 (Clone GK1.5 Cat# BE0003-1), both anti-CD4 and CD8, or isotype control (clone LTF-2 Cat# BE0090) on day −3, −1, and 1 of challenge. All mice were bled on day 1 post challenge via submandibular vein to confirm T-cell depletion by flow cytometry.

### Ferret vaccination and tissue collection

Male ferrets, 4 to 6 months old, were purchased from Triple F Farms (Gillett, PA) and determined to be seronegative to circulating seasonal influenza strains, by HI assay, and SARS-CoV-2, by ELISA. All animal experiments were approved and performed in accordance with St. Jude Children’s Research Hospital’s Animal Care and Use Committee protocol number 428. Ferrets were vaccinated intramuscularly, hind leg, with 0.1 mL volume of PBS, HAdV-5-Epigraph (5 × 10^10^ vp), or 0.5 mL FluZone® Quadrivalent Influenza Vaccine (2018–2019 Formula). All treatments were given in 2 doses, 3 weeks apart. Sera were collected 20 days post-vaccination (20 dpv) and 13 days post boost (B + 13) and assessed for antibody response by HI assay.

### Influenza virus challenges in ferrets

To evaluate protection, 2 weeks post boost, ferrets were anesthetized with isoflurane and inoculated intranasally with 1 mL of 10^5^ TCID_50_ of either H3N2 virus, A/Brisbane/10/07 or A/Hong Kong/2671/2019. Clinical signs of infection, weight loss, and temperature were monitored daily up to 14 days post challenge. On days 1, 3, 5, 7, and 9 after virus inoculation, ferrets were anesthetized with ketamine, and nasal washes were collected in 1 mL of phosphate-buffered saline (PBS). Two ferrets per group were euthanized on day 7 with lung and nasal turbinate specimens collected for histology. Nasal wash samples were titrated on MDCK cells and viral titer was determined.

### Pathology methods

The lungs and nasal mucosa were fixed via intratracheal/intranasal infusion and then continued immersion in 10% buffered formalin solution. Tissues were routinely processed and embedded in paraffin, sectioned, and stained with hematoxylin and eosin, with serial sections subjected to antigen retrieval for 30 min at 98 °C before undergoing immunohistochemical labeling of viral antigen using a primary goat polyclonal antibody (US Biological, Swampscott, MA) against influenza A, USSR (H1N1) at 1:1000 and a secondary biotinylated donkey anti-goat antibody (catalog number sc-2042; Santa Cruz Biotechnology, Santa Cruz, CA) at 1:200 on tissue sections.

### Hemagglutination inhibition assay

Sera from mice and ferrets were incubated with receptor destroying enzyme (RDE; (370013; Denka Seiken) at a 1:3 ratio (sera: RDE) overnight at 37 °C. The RDE was inactivated at 56 °C for 30 min. Sera were diluted to a starting ratio of 1:10 in DPBS and serially diluted two-fold in a 96-well V-bottom plate. An equal volume (25 μL) of 4 HAU of the virus was added to each well and the plate was incubated at room temp for 1 hr before the addition of 50 μL of 0.5% chicken red blood cells to each well. The hemagglutination patterns were read after 30 min.

### ELISpot assay

An IFNγ ELISpot assay was used to analyze the T-cell response after vaccination. Total T cells were examined using pooled peptides from peptide arrays of the HA protein of A/Uruguay/716/2007 (NR-18968), A/New York/384/2005 (NR-2603), and A/Perth/16/2009 (NR-19266). In addition, peptide arrays of the HA protein of influenza virus strains Texas/1977, Mississippi/1985, and Aichi/1968 were synthesized by GenScript and were 17-mers with 12 amino acid overlap. Polyvinylidene 96-well difluoride-backed plates (MultiScreen-IP, Millipore) were coated with 50 μl of anti-mouse IFN-γ mAb AN18 (5 µg/mL; Mabtech) overnight at 4 °C. Wells were then washed and blocked with cRPMI 10% FBS for 1 hr at 37 °C. A single-cell suspension of mouse splenocytes was added to each well. Splenocytes were re-stimulated with an equal volume (50 μL) of peptide (5 μg/mL/peptide) and incubated overnight at 37 °C with 5% CO_2_ to allow for IFNγ production. The next day, plates were washed 6× with PBS and incubated with 50 μL of biotinylated anti-mouse IFN-γ R4-6A2 mAb (1:1000 dilution; Mabtech) diluted in PBS with 1% FBS for 1 h at RT. Plates were washed 6× with PBS and incubated with 50 µl of streptavidin-alkaline phosphatase conjugate (1:1000 dilution; Mabtech) diluted in PBS 1% FBS. After 1 h at RT, the plates were washed 6× with PBS and developed by adding 100 µl of BCIP/NBT (Plus) alkaline phosphatase substrate (Thermo Fisher). Development was stopped by washing several times in dH_2_O. The plates were air-dried and spots were counted using an automated ELISpot plate reader (AID iSpot Reader Spectrum). Results are expressed as spot-forming cells per 10^6^ splenocytes.

### Tissue-culture infectious dose (TCID_50_)

Lung supernatant from day 3 post challenge was diluted 1:10 in a 96-well U bottom tissue-culture dish and serially diluted 10-fold. MDCK cells (100 μL of 2 × 10^5^ cells/mL) were added to each well and plates were incubated overnight at 37 °C with 5% CO_2._ The next day, plates were washed one time with sterile DPBS before adding DMEM with 0.0002% trypsin to each well. The plates were then incubated for another 3 days at 37 °C with 5% CO_2_ before adding 50 μL of 0.5% chicken red blood cells to each well and reading the hemagglutination patterns after 30 min.

### qPCR lung viral load quantification

RNA was extracted from day 3 post challenge lung supernatant using the PureLink Viral RNA/DNA Mini Kit according to the manufacturer’s instructions (Invitrogen). Real-time qPCR was performed using the Luna Universal Probe One-Step RT-qPCR Kit (NEB) run on a QuantStudio 3 Real-Time PCR System (Applied Biosystems) using the following cycling conditions: 55 °C for 30 min, 95 °C for 2 min, and 40 cycles of 95 °C for 15 s and 60 °C for 30 s. Results were compared to a standard curve created using a plasmid expressing the influenza matrix protein or RNA extracted from a known quantity of infectious Texas/1977 virus. The universal primer-probe set for Influenza A (BEI Resources, NR-15593, NR-15594, NR-15595) was used.

### Flow cytometry

To evaluate cytokine profiles, splenocytes from mice were harvested two weeks after boosting and stimulated for 10 h at 37 °C 5% CO_2_ with pooled A/Perth/16/2009 (NR-19266) peptides at 2 ug/mL with the addition of BD GolgiPlug (BD #555029). Cells were then stained with ZombieAqua Fixable cell viability dye (Biolegend #423101) according to the manufacturer’s instructions before Fc block with TruStain FcX™ (anti-mouse CD16/32) antibody (Biolegend #101319). Surface cell staining was performed with anti-CD8a-PerCP/Cyanine5.5 (Biolegend #100733), anti-CD4-PE/Cyanine7 (Biolegend #100421), anti-CD44-APC/Cyanine7 (Biolegend #103027), and CD3-PE (Biolegend #100205) for 30 min at 4 °C in the dark. Cells were then fixed and permeabilized with BD Cytofix/Cytoperm Plus Kit (BD #555028) according to the manufacturer’s instructions before intracellular cytokine staining with anti-IL-2-Brilliant Violet 421 (Biolegend # 503825), anti-IFN-γ-Alexa Fluor 488 (Biolegend #505815), anti-TNF-α-APC (Biolegend #506307), anti-IL-4-Brilliant Violet 605 (Biolegend # 504125) for 30 min at 4 °C in the dark. Cell events were acquired on a 4-laser/16-color Beckman Coulter CytoFLEX LX machine in the UNL Flow Cytometry Service Center Core and analyzed with FlowJo software. Cells were gated in the following order: lymphocytes > singlets > live > CD3^+^ > CD44^high^ > CD4^+^ or CD8^+^ followed by cytokine-producing cells (IFN-γ, IL-2, IL-4, and TNF-α). AbC™ Total Antibody Compensation Bead Kit was used to compensate the fluorophores.

To confirm T-cell depletion, whole blood was collected and red blood cells were lysed using ACK lysis buffer for 10 min. Cells were pelleted by centrifugation and then stained with CD4-FITC (#100509), CD3-PE (#100205), and CD8α-APC (#100711) for 30 min at 4 °C. Cell events were acquired on a 4-laser/16-color Beckman Coulter CytoFLEX LX machine in the UNL Flow Cytometry Service Center Core and analyzed with FlowJo software. All mice were confirmed to have greater than 98% depletion as compared to the isotype-treated mice.

### ELISA

Immunolon 4 HBX microtiter 96-well strips were coated with 150 ng per well of influenza A/Texas/1/1977 HA1 protein (Cat #: Cat: 40477-V08H1; SinoBiological) in bicarbonate/carbonate coating buffer overnight at 4 °C before blocking with 2% BSA in PBS for 2 h at RT. Serially diluted sera in 1% BSA in PBS was added to the plate and incubated for 2 h at RT before washing with PBST and incubation with secondary goat anti-mouse-HRP antibody (1:5000; Thermo Fisher) in 1% BSA in PBS for 1 h at RT. Plates were washed with PBS and developed with 1-Step Ultra TMB-ELISA (Thermo Fisher). The reaction was stopped with 2 M sulfuric acid and the OD450 was detected using a SpectraMax i3x Multi-Mode microplate reader (Molecular Devices). The endpoint titer was determined as a signal that was four times background values.

### Statistical analysis

GraphPad Prism 9 software was used to analyze all data. Data are expressed as the mean with standard error (SEM). T-cell data and lung viral titers were analyzed using one-way analysis of variance with Tukey’s multiple comparisons. HI titers were analyzed using unpaired T-tests. The significance of survival outcomes was analyzed by the Kaplan–Meier log-rank test. A *p* value < 0.05 was considered statistically significant.

### Reporting summary

Further information on research design is available in the Nature Research Reporting Summary linked to this article.

## Supplementary information

Supplemental Figures

Reporting Summary

## Data Availability

The Epigraph vaccine designer algorithm used in this study is freely available at https://www.hiv.lanl.gov/content/sequence/EPIGRAPH/Epigraph.html. All sequences used to create the Epigraph immunogens are freely available through the Influenza Research Database at https://www.fludb.org/brc/home.spg?decorator=influenza. All other relevant data will be provided by the corresponding author upon request.
