# Supplementary material for: An epitope-optimized human H3N2 influenza vaccine induces broadly protective immunity in mice and ferrets
Source: NPJ Vaccines. 2022 Jun 23;7:65. doi: 10.1038/s41541-022-00492-y (PMC9226166; doi:10.1038/s41541-022-00492-y)
Supplement: Supplementary file 1 — Supplemental Figures [file 41541_2022_492_MOESM1_ESM.pdf]

	% amino acid identity to			
	Epigraph1	Epigraph2	Epigraph3	A/Singapore/INFIMH-16-0019/2016
A/Hong Kong/2671/2019	95.9	90.5	88.3	97.5
A/Wisconsin/04/2018	97.7	92.4	88.3	97.9
A/Kansas/14/2017	97.3	90.6	88.3	97.0
A/Singapore/INFIMH-16-0019/2016	97.2	91.9	88.3	100.0
A/Alaska/232/2015	97.5	91.3	88.7	98.9
A/Hong Kong/4801/2014	98.4	92.2	88.7	98.4
A/Switzerland/9715293/2013	97.8	90.1	88.7	96.4
A/Ohio/02/2012	98.2	91.0	89.8	97.0
A/Victoria/361/2011	99.3	91.3	89.6	97.0
A/Rhode Island/01/2010	98.6	91.5	89.8	96.3
A/Perth/16/2009	98.2	91.9	89.9	95.8
A/Victoria/210/2009	97.9	91.9	89.8	95.8
A/Uruguay/716/2007	97.7	91.7	89.9	95.4
A/Brisbane/10/2007	97.9	91.9	90.1	95.2
A/Wisconsin/67/2005	97.0	91.3	90.5	94.4
A/New York/384/2005	97.2	91.5	90.5	94.4
A/Mississippi/1/1985	91.7	88.2	87.3	89.9
A/Texas/1/1977	89.6	86.6	86.4	87.8
A/Aichi/2/1968	86.2	85.2	83.9	85.2

Supp. Table 1. Percent identity between vaccine immunogens and the representative panel of H3 viruses. HA sequences were aligned using ClustalW on Geneious 11 and the percent identity was calculated for each vaccine immunogen and the viruses used in this study.

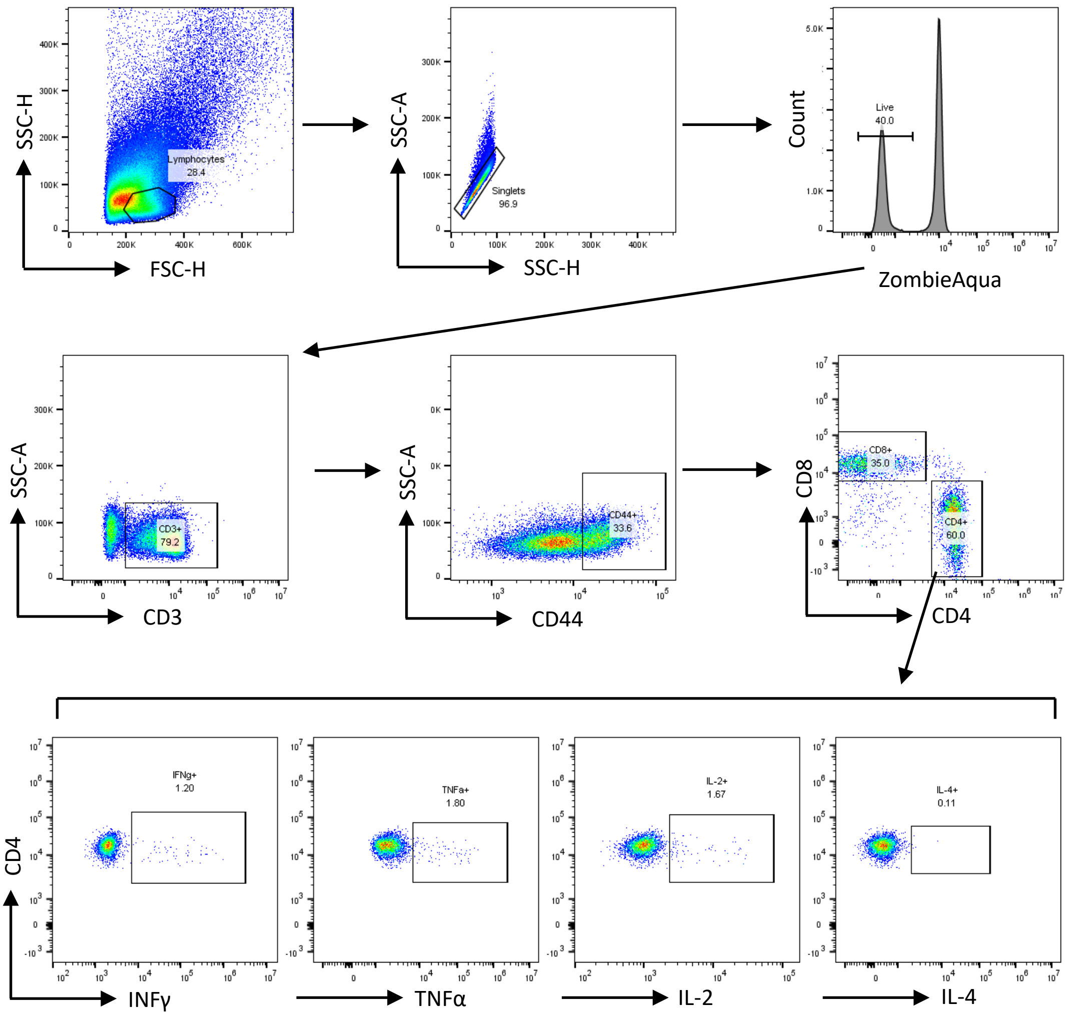

Supplemental Figure 1. Gating strategy for determining antigen-specific cytokine producing CD8+ and CD4+ T cells in vaccinated mice. Splenocytes from vaccinated BALB/c mice were harvested 2 weeks after boosting and stimulated with pooled Perth/2009 peptides before ICS and flow cytometry. Splenocytes were gated on the lymphocyte population, followed by singlet discrimination, and identification of live cells. Cells were then gated on $CD3^{+}CD44^{+}$ cells followed by either $CD4^{+}$ or $CD8^{+}$ cells. Cytokine producing cells were identified from both $CD4^{+}$ and $CD8^{+}$ populations and representative plots from of $CD4^{+}$ cells producing $IFNg^{+}$, $TNFa^{+}$, $IL\text{-}2^{+}$, and $IL\text{-}4^{+}$ are shown.

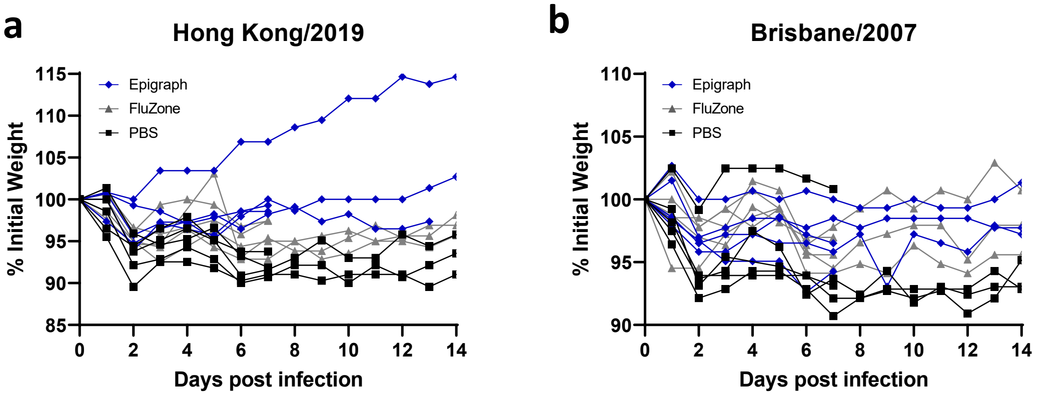

Supplemental Figure 2. Individual ferret weights post infection from Figure 8. The individual ferrets weeks for figure 8 after challenge with 10^5 TCID50 of Brisbane/2007 (a) or Hong Kong/2019 (d) (Day 1-7 n=5; Day 8-14 n=3).

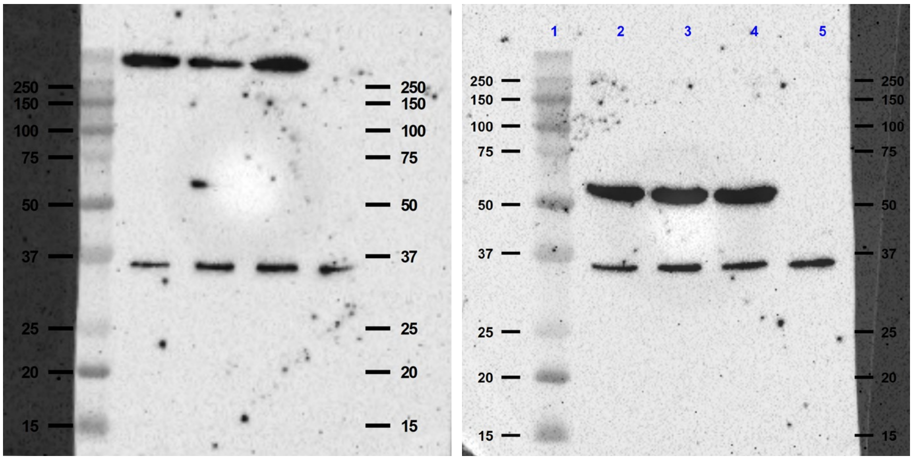

Supp. Figure 3. Full western blot images from Figure 1.
