# Supplementary material for: An epitope-optimized human H3N2 influenza vaccine induces broadly protective immunity in mice and ferrets
Source: NPJ Vaccines. 2022 Jun 23;7:65. doi: 10.1038/s41541-022-00492-y (PMC9226166; doi:10.1038/s41541-022-00492-y)
Supplement: Supplementary file 2 — Reporting Summary [file 41541_2022_492_MOESM2_ESM.pdf]

nature portfolio

Corresponding author(s): Dr Eric Weaver

Last updated by author(s): May 4, 2022

Reporting Summary

Nature Portfolio wishes to improve the reproducibility of the work that we publish. This form provides structure for consistency and transparency in reporting. For further information on Nature Portfolio policies, see our Editorial Policies and the Editorial Policy Checklist.

Statistics

For all statistical analyses, confirm that the following items are present in the figure legend, table legend, main text, or Methods section.

n/a	Confirmed	
☐	☒	The exact sample size (*n*) for each experimental group/condition, given as a discrete number and unit of measurement
☐	☒	A statement on whether measurements were taken from distinct samples or whether the same sample was measured repeatedly
☐	☒	The statistical test(s) used AND whether they are one- or two-sided<br>*Only common tests should be described solely by name; describe more complex techniques in the Methods section.*
☒	☐	A description of all covariates tested
☒	☐	A description of any assumptions or corrections, such as tests of normality and adjustment for multiple comparisons
☐	☒	A full description of the statistical parameters including central tendency (e.g. means) or other basic estimates (e.g. regression coefficient) AND variation (e.g. standard deviation) or associated estimates of uncertainty (e.g. confidence intervals)
☒	☐	For null hypothesis testing, the test statistic (e.g. *F*, *t*, *r*) with confidence intervals, effect sizes, degrees of freedom and *P* value noted<br>*Give P values as exact values whenever suitable.*
☒	☐	For Bayesian analysis, information on the choice of priors and Markov chain Monte Carlo settings
☒	☐	For hierarchical and complex designs, identification of the appropriate level for tests and full reporting of outcomes
☒	☐	Estimates of effect sizes (e.g. Cohen's *d*, Pearson's *r*), indicating how they were calculated

Our web collection on statistics for biologists contains articles on many of the points above.

Software and code

Policy information about availability of computer code

Data collection	The Epigraph Vaccine Designer from the Los Alamos National Laboratories was used to design the three H3 vaccine immunogens.
Data analysis	The resulting three HA Epigraph genes (Epigraph 1, 2, and 3) were added back to the H3 sequence population and aligned using ClustalW. The maximum likelihood phylogenetic tree was created using the RAxML-HPC BlackBox tool with a Jones-Taylor-Thornton substitution model using CIPRES Science Gateway V3.3 on the Extreme Science and Engineering Discovery Environment (XSEDE). The tree was visualized using the Geneious 11.1.5 software. The maximum likelihood phylogenetic tree to compared the assay strains to the vaccine strains was created using PhyML 3.3 with a Dayhoff substitution model on the Geneious 11.1.5 software.

For manuscripts utilizing custom algorithms or software that are central to the research but not yet described in published literature, software must be made available to editors and reviewers. We strongly encourage code deposition in a community repository (e.g. GitHub). See the Nature Portfolio guidelines for submitting code & software for further information.

Data

Policy information about availability of data

All manuscripts must include a data availability statement. This statement should provide the following information, where applicable:

- Accession codes, unique identifiers, or web links for publicly available datasets
- A description of any restrictions on data availability
- For clinical datasets or third party data, please ensure that the statement adheres to our policy

The Epigraph vaccine designer algorithm used in this study is freely available at https://www.hiv.lanl.gov/content/sequence/EPIGRAPH/Epigraph.html. All sequences used to create the Epigraph immunogens are freely available through the Influenza Research Database at https://www.fludb.org/brc/home.spg?decorator=influenza. All other relevant data will be provided by the corresponding author upon request.

Field-specific reporting

Please select the one below that is the best fit for your research. If you are not sure, read the appropriate sections before making your selection.

☒ Life sciences ☐ Behavioural & social sciences ☐ Ecological, evolutionary & environmental sciences

For a reference copy of the document with all sections, see nature.com/documents/nr-reporting-summary-flat.pdf

Life sciences study design

All studies must disclose on these points even when the disclosure is negative.

Sample size	We used sample sizes of 5 - 10 animals per group. The number of animals is clearly described in the methods and figure legends. A sample size of 5 is sufficient to reach statistical significance.
Data exclusions	No data were excluded.
Replication	In many cases the experiments were successfully replicated. In addition, we used two animal models that effectively show very similar results in both immune correlates and challenge studies.
Randomization	No randomization needed
Blinding	Blinding was not necessary since experiments were confirmed in two separate laboratories.

Reporting for specific materials, systems and methods

We require information from authors about some types of materials, experimental systems and methods used in many studies. Here, indicate whether each material, system or method listed is relevant to your study. If you are not sure if a list item applies to your research, read the appropriate section before selecting a response.

Materials & experimental systems

n/a	Involved in the study	
☐	☒	Antibodies
☐	☒	Eukaryotic cell lines
☒	☐	Palaeontology and archaeology
☐	☒	Animals and other organisms
☒	☐	Human research participants
☒	☐	Clinical data
☒	☐	Dual use research of concern

Methods

n/a	Involved in the study	
☒	☐	ChIP-seq
☐	☒	Flow cytometry
☒	☐	MRI-based neuroimaging

Antibodies

Antibodies used	anti-HA Tag HRP conjugated antibody (NB600-391; Novus Biologicals), anti-GAPDH (sc-47724), goat anti-mouse HRP conjugated antibody (Millipore Sigma #AP308P), primary goat polyclonal antibody (US Biological, Swampscott, MA) against influenza A, USSR (H1N1), biotinylated donkey anti-goat antibody (catalog number sc-2042; Santa Cruz Biotechnology, Santa Cruz, CA), anti-mouse IFN-γ mAb AN18 (5 μg/mL; Mabtech), biotinylated anti-mouse IFN-γ R4-6A2 mAb (Mabtech), with TruStain FcX™ (anti-mouse CD16/32) antibody (Biolegend #101319), anti-CD8a-PerCP/Cyanine5.5 (Biolegend #100733), anti-CD4-PE/Cyanine7 (Biolegend #100421), anti-CD44-APC/Cyanine7 (Biolegend #103027), CD3-PE (Biolegend #100205), anti-IL-2-Brilliant Violet 421 (Biolegend # 503825), anti-IFN-γ-Alexa Fluor 488 (Biolegend #505815), anti-TNF-α-APC (Biolegend #506307), anti-IL-4-Brilliant Violet 605 (Biolegend # 504125), CD4-FITC (#100509), CD3-PE (#100205), and CD8α-APC (#100711), goat anti-mouse-HRP antibody (1:5000; Thermo Fisher), anti-mouse CD8α (Clone 2.43 Cat# BE0061), and anti-mouse CD4 (Clone GK1.5 Cat# BE0003-1)
Validation	All antibodies were supplied by commercial vendors or repositories and include quality control assessments.

Eukaryotic cell lines

Policy information about cell lines

Cell line source(s)	293 cells were received from Microbix and the MDCK cells were received from the International Reagent Resource.
Authentication	We did not authenticate since we used commercial or repositories that include quality control and verification.
Mycoplasma contamination	Cells were not screened for mycoplasma since they were obtained from commercial sources or repositories.

Commonly misidentified lines (See ICLAC register)	None

Animals and other organisms

Policy information about studies involving animals; ARRIVE guidelines recommended for reporting animal research

Laboratory animals	Female BALB/c mice ages 6-8 weeks were purchased from Jackson Laboratory. Male ferrets, 4 to 6 months old, were purchased from Triple F Farms (Gillett, PA)
Wild animals	N/A
Field-collected samples	N/A
Ethics oversight	All animal studies were approved by institutional animal care and use committees as described in the manusript. The protocols were approved by the UNL Institutional Animal Care and Use Committee (IACUC) (Project ID 1717) and the St. Jude Children's Hospital IACUC (Project ID 428).

Note that full information on the approval of the study protocol must also be provided in the manuscript.

Flow Cytometry

Plots

Confirm that:

☒ The axis labels state the marker and fluorochrome used (e.g. CD4-FITC).

☒ The axis scales are clearly visible. Include numbers along axes only for bottom left plot of group (a 'group' is an analysis of identical markers).

☒ All plots are contour plots with outliers or pseudocolor plots.

☒ A numerical value for number of cells or percentage (with statistics) is provided.

Methodology

Sample preparation	To evaluate cytokine profiles, splenocytes from mice were harvested two weeks after boosting and stimulated for 10 hrs at 37°C 5% CO2 with pooled A/Perth/16/2009 (NR-19266) peptides at 2 ug/mL with the addition of BD GolgiPlug (BD #555029).
Instrument	Cell events were acquired on a 4-laser/16 color Beckman Coulter CytoFLEX LX machine in the UNL Flow Cytometry Service Center Core and analyzed with FlowJo software.
Software	FlowJo software.
Cell population abundance	Flow cytometry was performed using ≥10,000 events.
Gating strategy	Gating strategy for determining antigen-specific cytokineproducing CD8+ and CD4+ T cells in vaccinated mice. Splenocytes from vaccinated BALB/c mice were harvested 2 weeks after boosting and stimulated with pooled Perth/2009 peptides before ICS and flow cytometry. Splenocytes were gated on the lymphocyte population, followed by singlet discrimination, and identification of live cells. Cells were then gated on CD3+ CD44+ cells followed by either CD4+ or CD8+ cells. Cytokine producing cells were identified from both CD4+ and CD8+ populations and representative plots from CD4+ cells producing IFNg+, TNFa+, IL-2+, and IL-4+ are shown.

☒ Tick this box to confirm that a figure exemplifying the gating strategy is provided in the Supplementary Information.
